# Securing Smart Grid Enabled Home Area Networks with Retro-Reflective Visible Light Communication

**DOI:** 10.3390/s23031245

**Published:** 2023-01-21

**Authors:** Mathew Salas, Sihua Shao, Adrian Salustri, Zachary Schroeck, Jun Zheng

**Affiliations:** 1 Department of Electrical Engineering, New Mexico Tech, Socorro, NM 87801, USA; 2 Department of Computer Science, Montana Technological University, Butte, MT 59701, USA; 3 Department of Computer Science & Engineering, New Mexico Tech, Socorro, NM 87801, USA

**Keywords:** visible light communication, heterogeneous network, backscatter communication, retro-reflective, smart grid, home area networks, key exchange, security

## Abstract

Smart appliances’ run schedule and electric vehicles charging can be managed over a smart grid enabled home area network (HAN) to reduce electricity demand at critical times and add more plug-in electric vehicles to the grid, which eventually lower customers’ energy bills and reduce greenhouse gas emissions. Short range radio-based wireless communication technologies commonly adopted in a HAN are vulnerable to cyber attacks due to their wide interception range. In this work, a low-cost solution is proposed for securing the low-volume data exchange of sensitive tasks (e.g., key management and mutual authentication). Our approach utilizes the emerging concept of retro-reflector based visible light communication (Retro-VLC), where smart appliances, IoT sensors and other electric devices perform the sensitive data exchange with the HAN gateway via the secure Retro-VLC channel. To conduct the feasibility study, a multi-pixel Retro-VLC link is prototyped to enable quadrature amplitude modulation. The bit error rate of Retro-VLC is studied analytically, numerically and experimentally. A heterogeneous Retro-VLC + WLAN connection is implemented by socket programming. In addition, the working range, sniffing range, and key exchange latency are measured. The results validate the applicability of the Retro-VLC based solution.

## 1. Introduction

Smart grids are modern electricity distribution systems that monitor, protect, and automatically optimize the operation of interconnected elements including generation equipment, high-voltage distribution, automation systems, and energy storage [1]. As a subset of smart grids, smart microgrids operate in a grid-connected mode and offer the benefits of distributed computing and communications to deliver real-time information and enable the instantaneous balancing of electrical supply and demand at the level required for each discrete device [2]. The ability of smart microgrids to deliver electricity to loads in a home area network (HAN) on a targeted, as needed basis is important to their success. Internal access to HAN energy management systems enables microgrid operations to perform multi-tier custom diagnostics and make selective decisions to schedule load shedding and level demand in real time. Electricity loads (consuming devices) can be divided into groups based on the degree of need for electricity. They are commonly categorized as sensitive, adjustable, or sheddable [2]. Tier 1 loads (sensitive) are those that must operate continuously without fail, such as elevators, refrigeration equipment, and emergency lighting. Tier 2 loads are discretionary (adjustable) and may be shifted or shed for short periods to balance generation availability. Examples include domestic water heating systems, certain fans, and air conditioning loads. Tier 3 loads (sheddable) are those that can be shed for emergency operations due to unplanned and partial loss of generation. Some loads may fall into different tier classifications based on the season or time of day. Inside a smart home, a HAN connects different tier loads including smart appliances, IoT sensors, and other electric devices to the energy management system of a smart grid through a HAN gateway (or smart meter). Smart appliances and devices will adjust their run schedule to reduce electricity demand on the grid at critical times and lower consumers’ energy bills [3]. The charging of a plug-in electric vehicle can also be managed over a HAN, e.g., bidirectional charging and electric vehicles for mobile storage [4]. The HAN can balance the demand of electricity across the household and prioritize between electric vehicles and other appliances to manage electricity usage and reduce costs. By adding more plug-in electric vehicles to the grid, we have the potential to reduce fuel costs, lower our dependency on foreign oil, and help reduce greenhouse gas emissions [3].

Short range radio-based wireless communication technologies, such as Zigbee, Bluetooth and WiFi, are existing candidates in a HAN that support two-way communication between the smart appliances, IoT sensors, electric vehicles, and the gateway [5]. However, this comes with a price as omnidirectional radio signals are vulnerable to intentional interceptions. Common cyber attacks in a wireless HAN include passive attack, masquerading, replay attack, denial of service (DOS) attack, and man-in-the-middle (MITM) attack [6]. Some of the attacks impose threats on individual household user privacy, while other attacks may even cause a widespread blackout by gaining control of portions of the grid. Although each wireless candidate has its unique security concerns (e.g., Zigbee shares the key with appliances, Bluetooth places the device into discoverable mode, and WiFi garners considerable interest from the hacker community), one common feature of these radio-based technologies causing the vulnerabilities is their wide interception range. Without sophisticated methods to protect the encryption keys, the data communication through the wireless channels are completely exposed to the public. In a WiFi-based HAN, pre-shared key WPA and WPA2 remain vulnerable to password cracking attacks [7]. Once adversaries discover the pre-shared key (PSK), they can potentially decrypt all packets encrypted with the PSK. Although more advanced 802.1X authentication [8] provides a stronger key protection, it requires a Remote Authentication Dial-In User Service (RADIUS) server and possibly also an Active Directory server, which will be costly for residential and small business settings.

**Figure 1:**
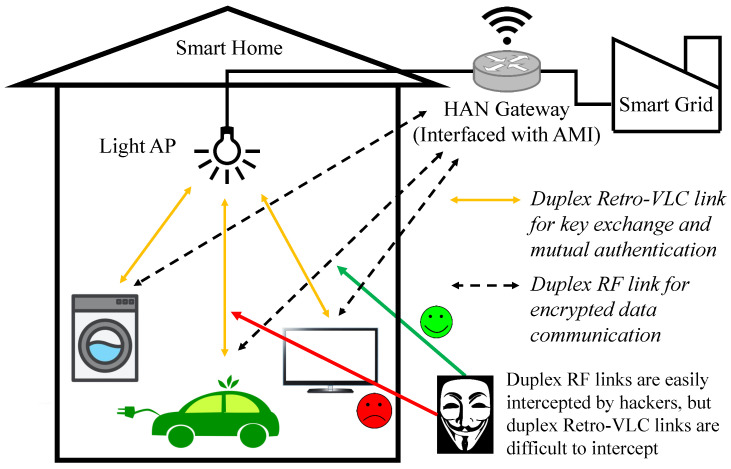
System architecture of heterogeneous RF and Retro-VLC home area networks.

**Figure 2:**
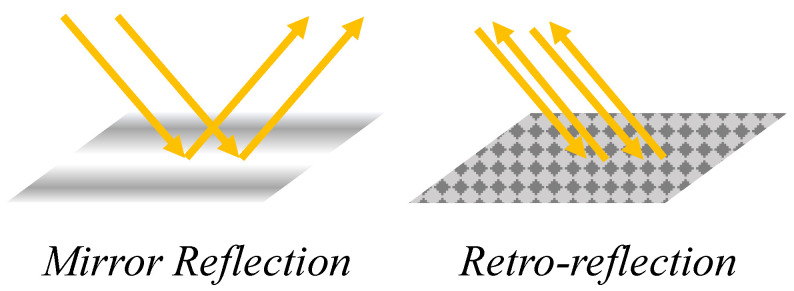
Mirror Reflection vs. Retro-reflection.

In this paper, we propose a heterogeneous radio frequency (RF) and Retro-VLC system (Figure 1) to secure wireless communication between smart appliances and IoT sensors in a HAN and the HAN gateway interfaced with the advanced metering infrastructure (AMI) of a smart grid. As shown in Figure 1, each smart appliance or IoT sensor in a HAN connects to the HAN gateway through two different wireless channels—a duplex Retro-VLC link for data exchange of sensitive tasks, e.g., key exchange, mutual authentication, association process; and a duplex RF link for transmitting and receiving data encrypted by the key shared through the Retro-VLC link. The study in [9] validates the feasibility of eavesdropping on a VLC signal, while intercepting both the VLC downlink and the Retro-VLC uplink is very difficult to achieve. The Retro-VLC link is established by interfacing retro-reflective tags with smart appliances and IoT sensors in a HAN. Optical modulator, e.g., liquid crystal shutters, are mounted on top of the retro-reflective tags to modulate the uplink data transmission from the tags to the lighting infrastructure. The Retro-VLC solution is adopted due to its extremely narrow interception range. As shown in Figure 2, different from a mirror reflection, retro-reflection reflects the incident light beams back to its source with minimal scattering. Replacing the low-cost retro-reflective film [10] with an accurately calibrated corner-cube retro-reflector [11] array can further reduce the scattering effect of the retro-reflection such that the possible sniffing range will be narrowed down to the line-of-sight between the tag and the reader.

To study the feasibility of the proposed heterogeneous Retro-VLC + WLAN system, a multi-pixel Retro-VLC prototype is designed and implemented. As the optical intensities from the individual pixels constructively add up, the effective retro-reflected intensity is generated by the sum of all intensities, such that the effective signal is constructed over the optical uplink channel but not in the electrical domain. The multi-pixel mode supports more spectral-efficient modulation schemes, e.g., orthogonal frequency division multiplexing (OFDM), than on-off keying (OOK) in the single-pixel mode. Due to the digital formation process, the generated sinusoidal signal through the multi-pixel design is imperfect. We investigate the non-idealities using a quadrature amplitude modulation (QAM) signal and its corresponding I-Q constellation diagram. The distortion of a QAM signal is quantified based on the relationship between the number of pixels and the QAM signal’s amplitude and phase offsets in the I-Q plane. The bit error rate (BER) performance of the Retro-VLC link is studied in simulations and experiments, and integrated into a heterogeneous network configuration for the evaluation of working range, sniffing range, and key exchange latency. The results demonstrate the suitability of duplex Retro-VLC link with respect to securing low-volume sensitive data exchange.

The manuscript is organized as follows: in Section 2, we derive a close-form expression of BER for multi-pixel Retro-VLC and build on the architecture of a heterogeneous Retro-VLC + WLAN system and the testbed setup. Simulation and experimental results are presented in Section 3 to evaluate the BER performance, key exchange latency, and working and sniffing range. We discuss the findings, implications and future research challenges in Section 4.

## 2. Methodology

In this section, we quantitatively study the impact of multi-pixel design on the I-Q offset and BER performance, elaborate the architecture of the heterogeneous Retro-VLC + WLAN system, and also demonstrate the testbed setup of Retro-VLC link and heterogeneous connection.

### 2.1. BER Analysis for Multi-pixel Design

**Figure 3:**
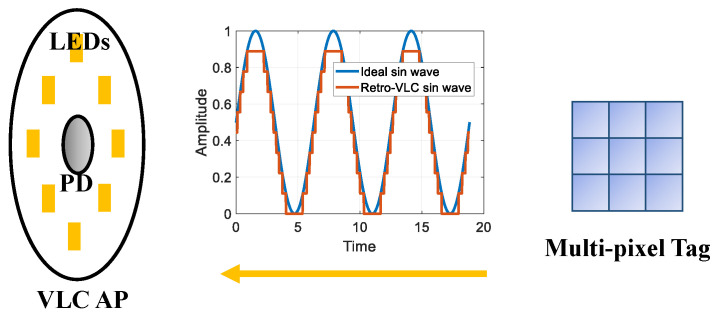
Signal distortion caused by multi-pixel design in Retro-VLC system.

As shown in Figure 3, the multi-pixel tag reflects the downlink light from the VLC access point (AP) back to the photodiode (PD) embedded on the VLC AP, and modulates the uplink optical signal through separate control of each individual pixel. A multi-level signal generated by the multi-pixel design can be utilized to emulate a sinusoidal wave and thus enables more spectral-efficient modulation schemes than OOK in the single-pixel mode. The distortion of the sinusoidal wave is determined by the number of pixels. In this subsection, we quantify the impact of the distortion on the amplitude and phase offsets of a general M-QAM signal and use 4-QAM as a paradigm to discuss the effect on BER performance.

With *n* equally sized pixels, n+1 amplitude values can be attained. A quantized arbitrary waveform can be constructed using the n+1 signal levels. Consider an arbitrary signal s(t) within the range [−a,a]; the quantized s(t) can be computed as follows:

(1) $$Q\left[s\left(t\right)\right]=\left\lfloor \frac{n(s(t)+a)}{2a}\right\rceil .$$

The right-hand side of equation first changes the signal range from [−a,a] to [0,n] and then rounds each value to the nearest integer. Each integer level corresponds to a certain number of pixels during the process of signal generation.

In general, a QAM signal can be written as [12]

(2) s(t)=Re{u(t)e2πjfct},

where $u\left(t\right)=x_{I}\left(t\right)+jx_{Q}\left(t\right)$ is a complex waveform with the real part $x_{I}\left(t\right)$ representing the inphase amplitude and imaginary part $x_{Q}\left(t\right)$ representing the quadrature amplitude; $f_{c}$ is the carrier frequency. A noiseless channel is assumed to achieve isolation of the I-Q offset caused by additive white Gaussian noise (AWGN). The main noise factors at the optical receiver are shot noise and thermal noise [13,14,15]. In most of the optical wireless channel literature [13,14,15], both the shot noise and the thermal noise are modeled as AWGN.

Thus, in the analysis, the noiseless received signal r(t) can be represented by Q[s(t)]. To demodulate the received signal, we first convert the signal range back to [−a,a] from [0,n], and then multiply by $e^{-2\pi jf_{c}t}$ to separate the inphase and quadrature amplitudes into the real and imaginary parts. Therefore, the demodulated signal can be represented by

(3) $$\hat{r}\left(t\right)=\left(\frac{2a}{n}Q\left[s\left(t\right)\right]-a\right)e^{-2\pi jf_{c}t}.$$

For a noiseless channel, the sampled values of $\hat{r}\left(t\right)$ at $\frac{1}{2f_{c}}$ and $\frac{1}{4f_{c}}$ generate the I and Q outputs, respectively, by forcing the double-frequency terms to be zero. Therefore, to quantify the I-Q offset, we derive the close-form expressions for $\hat{r}\left(\frac{1}{2f_{c}}\right)$ and $\hat{r}\left(\frac{1}{4f_{c}}\right)$.

**Lemma** **1.**
$Q\left[-s\left(t\right)\right]-\frac{n}{2}=-Q\left[s\left(t\right)\right]+\frac{n}{2}$

**Proof.**  The proof of Lemma 1 is provided in Appendix A. □

**Theorem** **1.** *For a demodulated signal $\hat{r}\left(t\right)=\left(\frac{2a}{n}Q\left[s\left(t\right)\right]-a\right)e^{-2\pi jf_{c}t}$, the I output is r^(12fc)=2anQ[Re{u(t)}]−a, and the Q output is r^(14fc)=j(2anQ[Im{u(t)}]−a).*

**Proof.**  The proof of Theorem 1 is provided in Appendix B. □

For 4-QAM, if we denote the transmitted inphase and quadrature amplitudes both as $\sqrt{E_{b}}$, according to Theorem 1, the I-Q outputs will be shifted to $\frac{2a}{n}Q\left[\sqrt{E_{b}}\right]-a$. Therefore, an I-Q offset ΔV can be defined as

(4) $$\Delta V={\left(\frac{2a}{n}Q\left[\sqrt{E_{b}}\right]-a\right)}^{2}-E_{b}.$$

The BER equation for 4-QAM without I-Q offset is $\frac{1}{2}erfc\sqrt{\frac{E_{b}}{N_{0}}}$ [12]. $E_{b}$ denotes the distance between the four I-Q points and the original point of the constellation diagram. Since ΔV provides a further I-Q offset, it changes the numerator part of the BER equation to $E_{b}+\Delta V$. Therefore, the BER equation for 4-QAM with I-Q offset is

(5) $$BER=\frac{1}{2}erfc\left(\sqrt{\frac{E_{b}+\Delta V}{N_{0}}}\right).$$

In Section 2.3.1, we further compare the analytical results with the simulation and experimental results to validate the proof of Theorem 1.

### 2.2. Heterogeneous Retro-VLC and WLAN System

**Figure 4:**
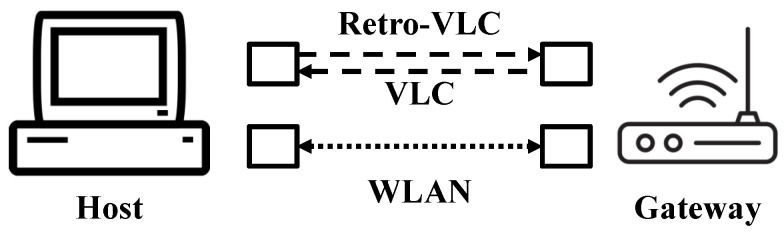
Architecture of heterogeneous Retro-VLC and WLAN system.

In the proposed heterogeneous Retro-VLC and WLAN system (Figure 4), each smart appliance or IoT sensor (denoted as host) in a smart-grid-enabled HAN is equipped with a hybrid network adapter, which enables a two-way communication between the device and the HAN gateway through two different wireless access technologies. The idea of connecting a client to the AP through two different channels originates from the concept of channel aggregation [16] (i.e., binding the two squares in Figure 4). This can be implemented on different layers of the OSI reference model, ranging from the data link layer to the application layer. Relying on higher layers requires considerable modifications at both the client and server sides, which prohibits large-scale deployment. On the other hand, lower layer channel aggregation can be implemented only at the client side with some adaptive changes at higher layers. In our heterogeneous testbed (Section 2.3.2), socket programming is adopted to gain complete control of the data link layer for packet-type-based link selection.

The architecture of the heterogeneous system consists of a Retro-VLC tag, a photodetector, and a WiFi transceiver on the host side, as well as a light source with a embedded PD, and a WiFi transceiver on the gateway side. The full-duplex VLC link is asymmetric, i.e., traditional VLC [13] is used to send downlink data from the gateway to the host and Retro-VLC is used to send uplink data from the host to the gateway. Retro-VLC uplink significantly reduces the interception range (experimentally evaluated in Section 3.3 and also provides several unique advantages, e.g., highly directional communication with narrow beam for reduced interference, dense spatial multiplexing for enhanced spectral efficiency, self-alignment for reliable connectivity, and μW power consumption for ultra-low power applications [17,18,19]. Nevertheless, the state-of-the-art optical modulator (i.e., liquid crystal shutter) has a limited modulation bandwidth which results in throughput up to several kbps [20]. Therefore, we consider the Retro-VLC uplink suitable for low-volume and sensitive data exchange. In Section 3.2, we experimentally study the key-exchange latency of the asymmetric VLC link.

### 2.3. Testbed Setup

#### 2.3.1. Retro-VLC Link

**Figure 5:**
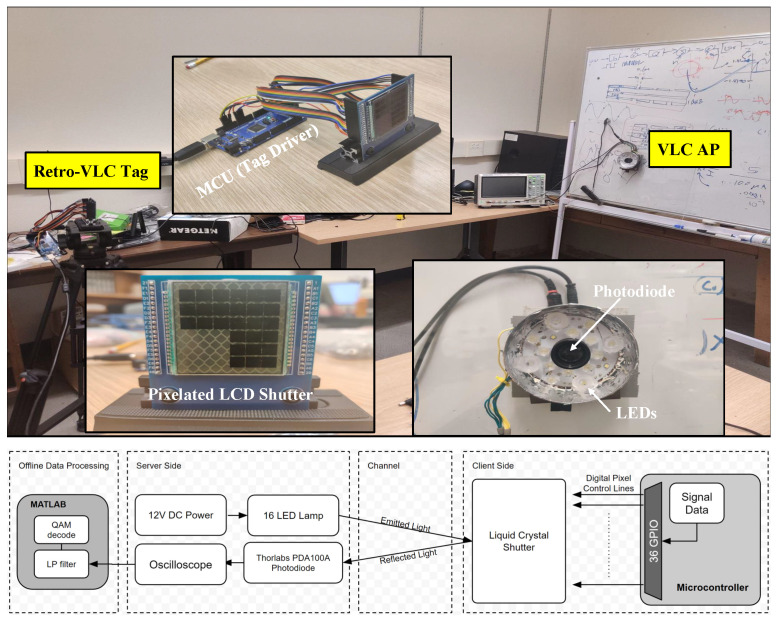
Testbed setup of Retro-VLC link.

The Retro-VLC testbed (Figure 5) includes a Retro-VLC tag with a microcontroller unit (MCU) and a pixelated liquid crystal display (LCD) shutter, a VLC AP with LEDs and an embedded PD, and a SDS2104X plus digital oscilloscope (sourced from SIGLENT Technologies, Solon, OH 44139, USA) for displaying and recording the data output from the VLC AP.

On the Retro-VLC tag, the LCD shutter is a customized 6x6 graphic shutter from liquid crystal technologies [21]. The size of each individual shutter is 6 mm × 6 mm. The LCD shutter covering a 3M retro-reflective tape [22] is mounted on a customized PCB board, which is used to wire the shutters to the I/O pins of the MCU. Each I/O pin runs a TTL logic signal to control its corresponding shutter with independent timing. A separated computer connected to the MCU is used to generate the QAM symbols. The symbol samples are sent over the serial port to the MCU. The MCU turns on and off I/O pins according to the received sample value. To avoid overflowing the serial port buffer, the computer sends new bytes only after receiving a request from the MCU.

On the VLC AP, the embedded PD is a silicon switchable gain detector PD100A2 from Thorlabs [23]. The gain is set to 20 dB. The PD is mounted with a 25 mm focal length lens in a 1″ diameter and 15 mm long lens tube. There are a total of 16 LEDs powered by a 12 V DC voltage source running through a 4 Ω resistor for current limiting. The output of the PD is fed directly into the oscilloscope with 2000 samples per second, which is much higher than the Nyquist rate. The recorded samples of the QAM signal are exported to Matlab for offline processing.

In addition to the demodulation procedures discussed in Section 2.1, the continuous dynamic phase offset [24] manifests as the points on the constellation diagram keep moving along the unit circle in the same direction and finally form a circle. The continuous dynamic phase offset is compensated for by multiplying each sample by a factor of $e^{\phi_{c}\times m}$, where *m* is an index of the sample being multiplied and $\phi_{c}$ is the difference in phase between consecutive QAM symbols. Hard-decision decoding [12] is adopted to convert the I-Q values into the received sequence of bits.

#### 2.3.2. Heterogeneous Connection

To ensure communication protocol compatibility, we use Ethernet connections to emulate the VLC and WLAN links. Linux wondershaper [25] is adopted to manually set the uplink and downlink speed of network interface cards (NICs) based on the throughput measurement of VLC and WLAN links. The heterogeneous testbed includes a host (two NICs), a switch, and a server (one NIC). Two Ethernet connections are set up between the host and the switch. One Ethernet connection is set up between the server and the switch. Using the socket programming written in C code (https://github.com/mathewsalas/Hetnet-Socket-Programming.git, accessed on 29 November 2022), the host binds two sockets to two Ethernet NICs, respectively, and the server binds one socket to its Ethernet NIC. The key exchange process is implemented by letting the server listen to its socket for a key request and sending the encryption key once an authorized request is received. The host sends a key request over the NIC emulating Retro-VLC uplink, receives an encryption key on the same NIC emulating the VLC downlink, and then applies the key to data encrpytion over the other NIC emulating the WLAN link. To evaluate the time cost of the key exchange process, we use the packet analyzer Wireshark on the host side to measure the time between the key request and the reception of the encryption key. According to the measurements based on the Retro-VLC testbed (Section 2.3.1), the data rates are set to 1 kbps and 10 Mbps for uplink and downlink data transmission, respectively.

## 3. Numerical and Experimental Results

### 3.1. BER Performance

To validate the BER (Equation (5)) derived in Section 2.1, we perform both numerical and experimental analysis for different numbers of pixels. Note that the total area of the reflecting surface of the pixelated shutter is fixed; as the number of pixels increases, the area of each pixel reduces. In BER experimental validation, to minimize the signal distortion caused by slow response of LCD shutter, we selected a low carrier frequency of 2 Hz.

**Figure 6:**
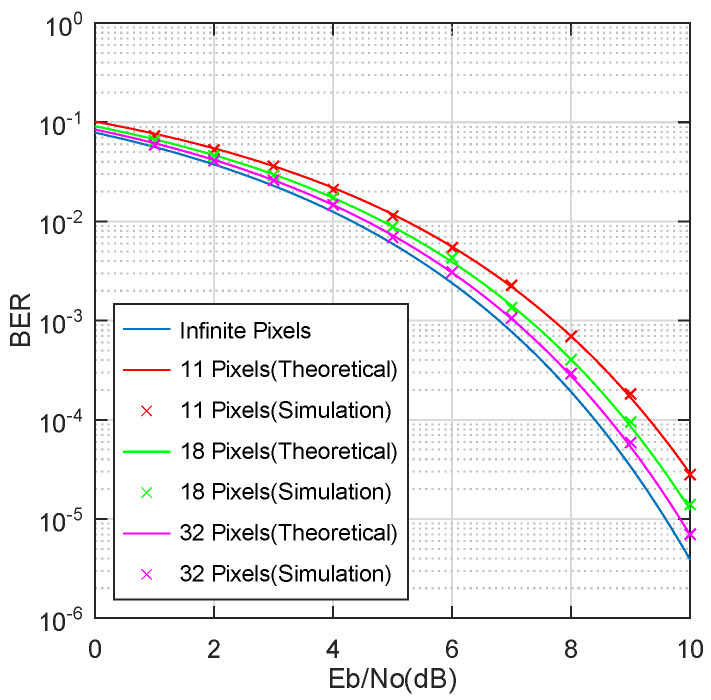
Theoretical results vs. simulation results for three different pixel numbers. The blue curve is plotted assuming an infinite number of pixels (i.e., ΔV=0).

In Figure 6, theoretical results are compared to simulation results for 11, 18, and 32 pixels. The lower bound of BER is calculated based on the assumption of an infinite number of pixels (i.e., ΔV=0). Different values of $\frac{E_{b}}{N_{0}}$ are considered to evaluate BER at different distances between the VLC AP and the Retro-VLC tag. In the simulation, 50,000 QAM symbols are generated randomly for each test when BER > $10^{-3}$, and the number of QAM symbols are increased to $10^{6}$ when BER < 10${}^{-3}$. The simulation was implemented in MATLAB R2020a. The simulator begins by generating a sampled time domain QAM signal at 100 samples per symbol. The samples are then quantized based on Equation (1). AWGN is superimposed on the quantized sample with a constant $\frac{E_{b}}{N_{0}}$. Finally, demodulation is performed in line with the procedures described in Section 2.1. We can observe from Figure 6 that as the number of pixels increases, the BER performance asymptotically approaches the lower bound (blue curve). The simulation results match well with the theoretical results with a mean square error of 6.4678%.

**Figure 7:**
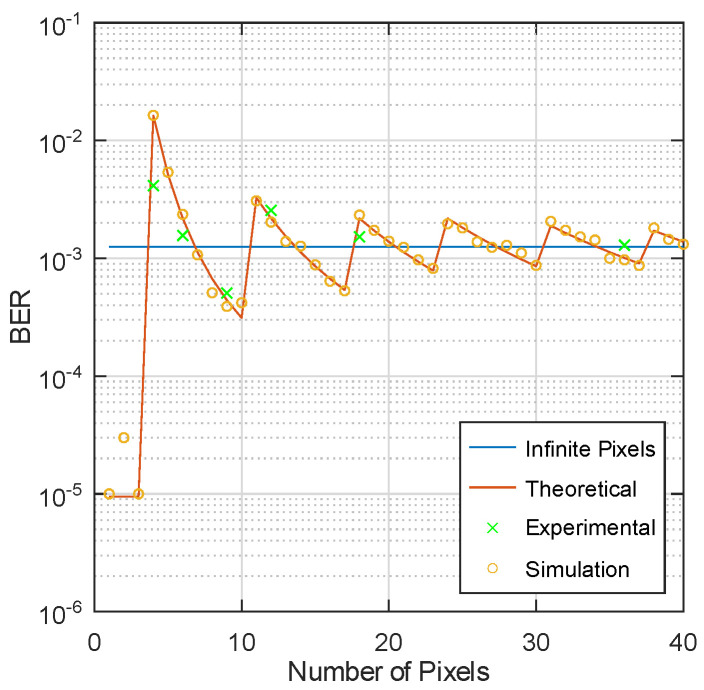
Theoretical results (red curve) generated by Equation (5), simulation results (yellow circle) and experimental results (green cross) are presented. The blue line is plotted assuming an infinite number of pixels (i.e., ΔV=0). $\frac{E_{b}}{N_{0}}$ is set at 6.6 dB based on experimental measurement.

In Figure 7, the AP-tag distance is fixed and the theoretical, simulation (https://github.com/mathewsalas/pixelated_RVLC_simulation.git, accessed on 30 November 2022) and experimental BER results are plotted as the number of pixels increases. The asymptotic line is also provided by assuming an infinite number of pixels. For experiments, to emulate a lower number of pixels, individual pixels are paired into blocks. Since each pixel covers the same area of the retro-reflective surface, each block can be treated as a larger single pixel. The graphic shutter used in Figure 5 has a total of 36 pixels; using the pairing method discussed above, the number of pixels we evaluate in experiments is the divisors of 36. The theoretical, simulated, and experimental results match well, and all exhibit non-monotonic behavior.

The sawtooth pattern of BER is the combinatorial result from the rounding operation and 4-QAM setting. First, the rounding operation in Equation (1) replaces the sample value by either a larger integer or a smaller integer depending on the number of pixels *n* and the signal amplitude *a*. The larger or smaller integer replacement will result in an outward I-Q shift (ΔV is positive) or an inward I-Q shift (ΔV is negative), respectively. Using hard decision decoding in 4-QAM, an outward I-Q shift will improve the BER performance, which is not necessarily true as the QAM order increases. With the number of pixels increased, the sign of ΔV will change multiple times, which is represented as the turning points of the BER curve.

### 3.2. Latency Evaluation

**Figure 8:**
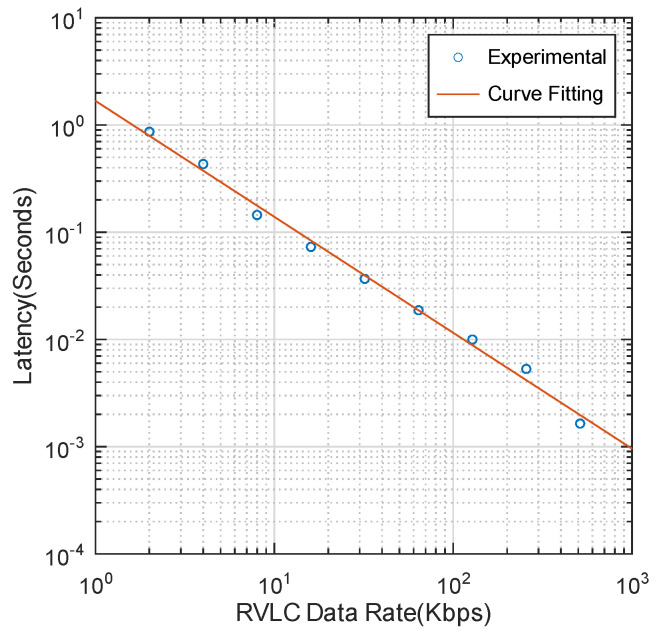
Retro-VLC uplink data rate vs key exchange latency showing the experimental results in blue and the curve fit used to extrapolate the expected time for other rates.

Due to the bandwidth limitation of the Retro-VLC uplink, the key exchange process may incur additional delay. To better understand the performance of the proposed heterogeneous network, experiments are conducted to measure the key exchange latency for different uplink data rates. The results are shown in Figure 8. To measure the time elapsed between the key request and the reception of the encryption key, we first use Linux Wondershaper [25] to manually set the uplink and downlink data rates for the purpose of emulating the Retro-VLC uplink and conventional VLC downlink. For each test, the downlink is always set to 10 Mbps and the uplink rate is changed from 2 Kbps to 1 Mbps. The key exchange process is initiated by a TCP three-way handshake, followed by a key request sent from the host to the gateway. The process is stopped with a key file of 100 bytes [26] sent from the gateway to the host, which includes the Pairwise Transient Key (PTK) and the Group Temporal Key (GTK) for unicast and multicast traffic encryption, respectively. Timestamps of all the transmitted and received TCP packets at the host side are captured by Wireshark and the key exchange latency is determined between the first and the last packets of the entire process. The results in Figure 8 exhibit an inverse proportional relationship between the uplink data rate and the key exchange latency. Using 4-QAM signals and unoptimized pixel configuration, the Pi-cell LCD shutter-based Retro-VLC tag can roughly achieve 1 Kbps uplink data rate. With data extrapolation, we can expect that the time cost of the key exchange at a 1 Kbps uplink rate is 1.6789 s. The latency could be significantly improved with a customized Retro-VLC medium access control (MAC) protocol and a larger bandwidth of optical modulators.

### 3.3. Working Range and Sniffing Range

**Figure 9:**
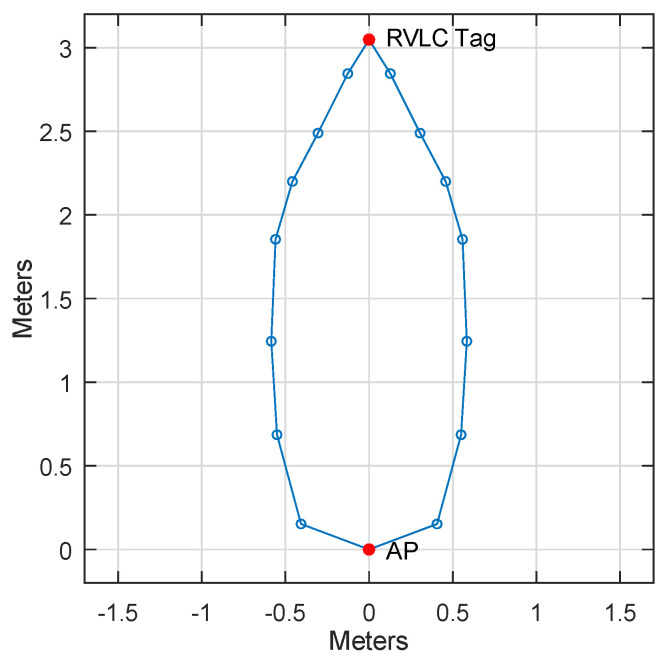
Working range.

We define the working range as the area within which the uplink BER is lower than 1%. We measure the working range in a lab environment, and show the results in Figure 9. The incidence angle and radiance angle are kept at the same value. The working range is delineated by the closed blue line. With an upright orientation of the Retro-VLC tag, the maximum working distance is 3 m. With the VLC AP perpendicular to the Retro-VLC tag plane, the field of view (FOV) is around 50°. The FOV can be improved by adding a diffusing layer on top of the VLC AP.

**Figure 10:**
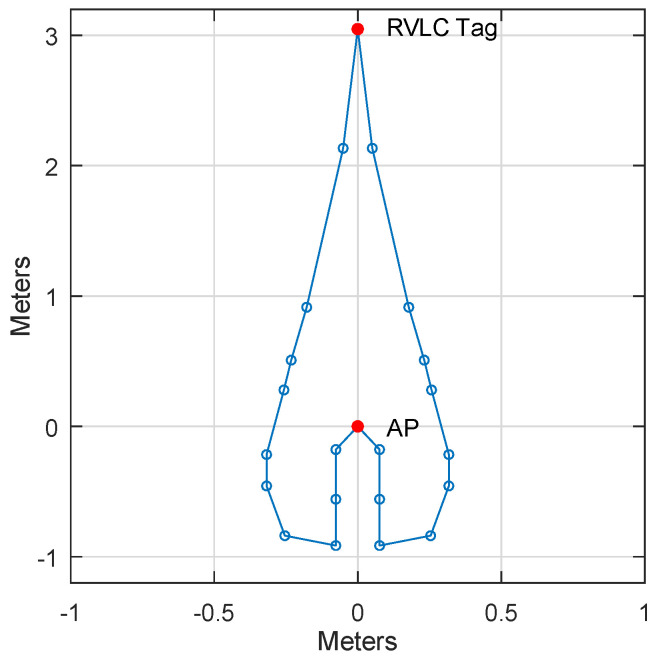
Sniffing range.

One key feature of Retro-VLC uplink when compared with radio-based wireless communication technologies is directional. In order to intercept the uplink Retro-VLC signal, the working locations (sniffing range) of an eavesdropping device are very limited [9]. In our evaluation, we place a VLC AP and a Retro-VLC tag 3 m apart from each other. The Retro-VLC tag is held upright and faces the VLC AP directly. We use the embedded PD as the sniffer and measure the range in which the sniffer can decode the uplink transmission with less than 1% BER. The area is plotted in Figure 10. We can observe that the shape of the possible sniffing area is a narrow region between the AP and the tag, plus a small region around the AP. The confined sniffing range protects the uplink transmission from typical cyber attacks, such as man-in-the-middle attack.

## 4. Discussion

In this work, the I-Q offset ΔV caused by the multi-pixel design of Retro-VLC is quantified with a closed-form expression (Equation (4)) and validated by simulation and experimental results (Section 3.1). The I-Q offset and the chosen modulation scheme have a combined effect on the final BER performance. Therefore, for a specific modulation scheme, an optimal number of pixels could be identified to minimize the BER. The bandwidth of the Retro-VLC tag could be significantly boosted to tens of kHz when considering a digital micro-mirror device (DMD) [27] instead of LCD shutter for the optical modulator. However, DMDs have a very limited field of view and do not inherently provide retro-reflection. In our future work, the BER analysis of multi-pixel design will be extended to OFDM modulation. Based on OFDMA, the concurrent BER performance of multiple Retro-VLC tags will be evaluated using our developed Retro-VLC testbed. The hardware design of the Retro-VLC will be optimized to further improve the uplink data rate.

## Data Availability

Not applicable.

## Appendix A. Proof of Lemma 1

**Proof.** By definition, $Q\left[-s\left(t\right)\right]=\left\lfloor \frac{-ns(t)}{2a}+\frac{n}{2}\right\rceil$ and $-Q\left[s\left(t\right)\right]=-\left\lfloor \frac{ns(t)}{2a}+\frac{n}{2}\right\rceil$. When *n* is even, $\frac{n}{2}$ is an integer, we have $\left\lfloor \frac{-ns(t)}{2a}+\frac{n}{2}\right\rceil =\left\lfloor \frac{-ns(t)}{2a}\right\rceil +\frac{n}{2}$. Given an arbitrary real number *x*, since ⌊−x⌉=−⌊x⌉, we have $\left\lfloor \frac{-ns(t)}{2a}\right\rceil +\frac{n}{2}=-\left\lfloor \frac{ns(t)}{2a}\right\rceil +\frac{n}{2}$ and $-\left\lfloor \frac{ns(t)}{2a}+\frac{n}{2}\right\rceil =-\left\lfloor \frac{ns(t)}{2a}\right\rceil -\frac{n}{2}$. Therefore, $Q\left[-s\left(t\right)\right]-\frac{n}{2}=-\left\lfloor \frac{ns(t)}{2a}\right\rceil +\frac{n}{2}-\frac{n}{2}=-\left\lfloor \frac{ns(t)}{2a}\right\rceil -\frac{n}{2}+\frac{n}{2}=-Q\left[s\left(t\right)\right]+\frac{n}{2}$. When *n* is odd, $\frac{n-1}{2}$ is an integer, we have $Q\left[-s\left(t\right)\right]-\frac{n}{2}=\left\lfloor \frac{n(-s(t)\;+\;a)}{2a}-\frac{n-1}{2}\right\rceil -\frac{1}{2}=\left\lfloor \frac{-ns(t)}{2a}+\frac{1}{2}\right\rceil -\frac{1}{2}$. Since $\left\lfloor x+\frac{1}{2}\right\rceil =\left\lceil x\right\rceil$, we have $\left\lfloor \frac{-ns(t)}{2a}+\frac{1}{2}\right\rceil -\frac{1}{2}=\left\lceil \frac{-ns(t)}{2a}\right\rceil -\frac{1}{2}$. $-Q\left[s\left(t\right)\right]+\frac{n}{2}=\left\lfloor \frac{-n(s(t)\;+\;a)}{2a}+\frac{n-1}{2}\right\rceil +\frac{1}{2}=\left\lfloor \frac{-ns(t)}{2a}-\frac{1}{2}\right\rceil +\frac{1}{2}$. Since $\left\lfloor x-\frac{1}{2}\right\rceil =\left\lfloor x\right\rfloor$, we have $\left\lfloor \frac{-ns(t)}{2a}-\frac{1}{2}\right\rceil +\frac{1}{2}=\left\lfloor \frac{-ns(t)}{2a}\right\rfloor +\frac{1}{2}$. Since $\left\lceil x\right\rceil -\frac{1}{2}=\left\lfloor x\right\rfloor +\frac{1}{2}$, we have $Q\left[-s\left(t\right)\right]-\frac{n}{2}=-Q\left[s\left(t\right)\right]+\frac{n}{2}$. □

## Appendix B

**Proof** **of Theorem 1.** For a noiseless channel, the I output r^(t)|t=12fc=(2anQ[s(t)]−a)e−2πjfct|t=12fc=(2anQ[Re{u(12fc)e2πjfc12fc}]−a)e−2πjfc12fc=(2anQ[Re{−u(12fc)}]−a)(−1). According to Lemma 1, we have (2anQ[Re{−u(12fc)}]−a)(−1)=−2an(Q[−Re{u(12fc}]−n2) = −2an(−Q[Re{u(12fc)}]+n2) = 2anQ[Re{u(12fc)}]−a. The Q output r^(t)|t = 14fc = (2anQ[Re{s(t)}]−a)e−2πjfct|t = 14fc = (2anQ[Re{u(14fc)e2πjfc14fc}]−a)e−2πjfc14fc=(2anQ[Re{u(14fc)j}]−a)(−j)=−2ajn(Q[Re{u(14fc)j}]−n2)=−2ajn(Q[−Im{u(14fc)}]−n2). According to Lemma 1, we have −2ajn(Q[−Im{u(14fc)}]−n2) = −2ajn(−Q[Im{u(14fc)}]+n2) = j(2anQ[Im{u(14fc)}]−a). □
